# An Evaluation of Agreement of Breathing Rates Measured by a Novel Device, Manual Counting, and Other Techniques Used in Clinical Practice: Protocol for the Observational VENTILATE Study

**DOI:** 10.2196/15437

**Published:** 2020-07-20

**Authors:** Matthew T Jones, Emily Heiden, Carole Fogg, Paul Meredith, Gary Smith, Nicola Sayer, Lisa Toft, Emma Williams, Matthew Williams, Thomas Brown, Jessica Gates, David Lodge, Paul Bassett, Mark Amos, Milan Chauhan, Selina Begum, Madeleine Rason, Jonathan Winter, Jayne Longstaff, Anoop J Chauhan

**Affiliations:** 1 Department of Respiratory Research and Innovation Portsmouth Hospitals NHS Trust Portsmouth United Kingdom; 2 Department of Nursing University of Bournemouth Bournemouth United Kingdom; 3 Portsmouth Hospitals Simulation Centre Portsmouth Hospitals NHS Trust Portsmouth United Kingdom; 4 Stats Consultancy Ltd Amersham United Kingdom; 5 Portsmouth Technology Trials Unit Portsmouth Hospitals NHS Trust Portsmouth United Kingdom

**Keywords:** respiratory, monitoring, device, medical, piezoelectric, respiration, vital signs, telemetry, respiratory function tests, tachypnoea, bradypnoea

## Abstract

**Background:**

Respiratory rate (RR) is the most sensitive physiological observation to predict clinical deterioration on hospital wards, and poor clinical monitoring has been highlighted as a primary contributor to avoidable mortality. Patients in intensive care have their RR monitored continuously, but this equipment is rarely available on general hospital wards.

**Objective:**

The primary objective is to assess the accuracy of the RespiraSense device in comparison with other methods currently used in clinical practice. The secondary objective is to assess the accuracy of the RespiraSense device in participants in different positions and when reading aloud.

**Methods:**

A single-center, prospective observational study will investigate the agreement of the RespiraSense device as compared with other device measurements (capnography, electrocardiogram) and the current standard measurement of RR (manual counting by a trained health care professional). The different methods will be employed concurrently on the same participant as part of a single study visit.

**Results:**

Recruitment to this study has not yet started as funding decisions are still pending. Therefore, results are not available at this stage. It is anticipated that the data required could be collected within 2 months of first recruitment to the study and data analysis completed within 6 months of the study start date.

**Conclusions:**

The Evaluation of Agreement of Breathing Rates Measured by a Novel Device, Manual Counting, and Other Techniques Used in Clinical Practice (VENTILATE) study will provide further validation of the use of the RespiraSense device in subjects with abnormal respiratory rates.

**International Registered Report Identifier (IRRID):**

PRR1-10.2196/15437

## Introduction

### Background

Patients admitted to acute hospitals undergo regular monitoring of their vital observations as a key component of their care. In 2007, the National Institute for Health and Clinical Excellence (NICE) produced recommendations for the measurement and recording of physiological observations [1] in response to several multicenter studies that revealed a significant lack of documentation of these observations.

Since the 1990s it has been recognized that the respiratory rate (RR) is the most sensitive physiological observation to predict cardiac arrest on hospital wards [2]. Despite this understanding, monitoring of the RR is demonstrated to be the most common vital sign to be inaccurately measured or recorded [3-5]. More recently, poor clinical monitoring has been highlighted as a primary contributor to avoidable mortality in English hospitals, implicated in 31% of preventable deaths [6].

The most commonly taught and used technique for measuring the RR is manually counting the number of breaths per minute a patient takes. A manual RR should be measured for a period of at least 1 minute, and the normal range for adults (aged 18 years and above) is 12 to 20 breaths per minute [7]. However, a recent cross-sectional study identified significant inaccuracies in the measurement of the RR among UK doctors and highlighted it as an important aspect of clinical care that is currently being poorly performed [8]. Manual counting among other clinical staff in routine practice has also been observed to be inaccurate, possibly due to the approach of counting breaths for 15 seconds and then multiplying by 4 to get breaths per minute, thus introducing error. This leads to somewhat of a self-fulfilling prophecy where expected inaccuracies in the measurement of the RR reduce the confidence of clinical staff at all levels in the usefulness of a reportedly abnormal RR [9].

Patients in intensive care are able to have their RR monitored accurately and continuously by capnography [10]. This type of monitoring can be invasive as it usually requires endotracheal intubation, and patients in other hospital areas do not usually need such an invasive intervention nor is the nursing staff likely to have access to such equipment. However, a systematic review has identified that the existing evidence for routine continuous noninvasive respiratory monitoring on general hospital wards is still inconclusive, and studies are lacking in methodological quality [11]. It recommends that future research should focus on technology explicitly suitable for general hospital wards and explore tailored alarm and treatment algorithms. The first step in such research is to explore the level of agreement between any new technology and current methods of establishing RR.

The significance of the accuracy of the RR recording cannot be overestimated [8,9]; it plays an important role in many clinical assessment systems, including the assessment of acute asthma and systemic inflammatory response syndrome and in the calculation of early warning scores [8]. This score alerts staff to any clinical deterioration and depending on the severity of the score, prompts a review from nursing or clinical staff.

The adoption of these scores has subsequently led to the development of multiple track and trigger information technology systems to allow early detection of patient deterioration [1]. Current systems rely on the manual assessment of the RR by a health care provider (HCP) and entry of data onto a live database in order for it to be processed. At Portsmouth Hospitals NHS Trust (PHT), the Vitalpac (System C) mobile clinical software system is used to record observations and calculates an early warning score (National Early Warning Score [NEWS]) depending on the degree of deviation from the normal range, with the total score proportionate to the overall level of risk (Table 1).

The RespiraSense device (PMD Solutions Ltd) was developed to improve the detection of changes in the RR in order to alert staff to out-of-range values that may be clinically significant. In a 48-patient investigation, data were collected to evaluate the difference in RR measurements between the developed RespiraSense device, electrocardiogram (ECG), and direct nursing observations [12]. A clinically relevant agreement between all RR measurements by the 3 different methods was demonstrated. However, few abnormal values were recorded in this investigation, and further research is required to ensure that the device works well to accurately measure the RR at values both higher and lower than the normal range.

**Table 1:** Escalation protocol for the deteriorating patient [8].

Vital sign	Score						
	3	2	1	0	1	2	3
Pulse	≤40	—	41-50	51-90	91-110	111-130	≥131 Unrecordable due to patient condition
Temperature (°C)	≤35	—	35.1-36	36.1-38	38.1-39	≥39	Unrecordable due to patient condition
BP^a^ systolic (mm Hg)	≤90	91-100	101-110	111-219	—	—	≥220 Unrecordable due to patient condition
Respiratory rate (bpm)	≤8	—	9-11	12-20	—	21-24	≥25 or Unrecordable due to patient condition
AVPU^b^	—	—	—	Alert	—	—	Voice pain unresponsive^c^
SaO_2_^d^	≤91	92-93	94-95	≥96 or Unrecordable because patient refused, equipment unavailable, other reason	—	—	Unrecordable due to patient condition
Inspired O_2_	—	—	—	Air	—	Any supplemental O_2_^e^	—

^a^BP: blood pressure.

^b^AVPU: alert, verbal, pain, unresponsive.

^c^If AVPU is V or C due to patient sedation, it will be charted as S and score 0 rather than 3.

^d^SaO_2_: oxygen saturation.

^e^Note that “any supplemental O_2_” applies to any supplemental oxygen the patient is receiving. It does not apply to patients who are on masks through which only air is being supplied (air delivery possible through tracheostomy, bilevel positive airway pressure or continuous positive airway pressure, for example).

### Objectives

This research aims to assess the accuracy of the RespiraSense device in comparison with other methods (capnography, ECG) and standard care (manual RR monitoring) in healthy participants across a range of predetermined RRs. The information from this study will contribute to the evidence base on RespiraSense to enable its use in clinical practice.

This investigation will supplement the following research that has been conducted in demonstrating the efficacy and/or accuracy of RespiraSense in a clinical setting:

- “Evaluation of a continuous monitoring device in capturing respiratory rate compared to industry standard and gold standard” (Bangor, study reference PMD-CS-007)
- “Can respiratory rate predict the risk of deterioration of septic patients?” (Denmark, study reference PMD-CS-006ii)
- “A quality assurance study of respiratory rate measurements on obese patients with a novel monitoring technology” (Portsmouth, study reference PMD-CS-011)

The primary objective is to assess the agreement of the RespiraSense device with alternative methods of RR counting in healthy participants. Secondary objectives are to (1) explore whether the position of the participant has any effect on the agreement between the RespiraSense device readings and alternative methods of RR counting, (2) explore whether the agreement of the RespiraSense device and alternative RR methods alters when a participant is reading aloud, and (3) establish agreement between manual counting of the RR and retrospective adjudication of video. The exploratory objective is to record any dermatological effects of the device.

## Methods

### Patient Selection

Selection criteria for the study are shown in Textbox 1.

**Selection criteria.**

**Inclusion**

- Male or female
- Aged ≥18 to 60 years
- Able and willing to provide written informed consent

**Exclusion**

- Any significant medical condition that may be worsened by the effect of slow or fast breathing
- Significant hearing impairment
- Skin unsuitable for device following assessment of skin fragility
- Unable to access left side of abdominal wall to attach device
- Neuromuscular disease and irregular chest wall movements
- Allergy to medical-grade skin adhesive
- Pregnant women during second and third trimester

### Recruitment

Participants will be recruited from the population of staff and students currently working or on placement at PHT. Flyers advertising the study will be posted in communal areas where these groups are likely to congregate (eg, doctors’ mess, ward break rooms). These flyers will contain contact details for the research team and advise those who are interested in taking part in the study to contact the research team.

Potential participants will be contacted by telephone and sent a participant information sheet (PIS). Participants who then wish to take part in the study will be invited to a screening visit. During this visit, the study will be explained, all questions answered, and participant will be screened against the inclusion and exclusion criteria. Additionally, the participant will be asked to breathe at a rate of 30 breaths per minute to assess their comfort while performing a study procedure. Those who are unable to maintain a rate of 30 breaths per minute for 2 minutes will be excluded from the study. Following this screening visit, the participant will be followed up with a telephone call to confirm that they are still willing to take part in the study.

### Study Design

The study is a single-center, prospective observational study to investigate the agreement of the RespiraSense device compared with (1) other device measurements (capnography, ECG) and (2) the current standard measurement of RR (manual counting by a trained HCP). The different methods will be employed concurrently on the same participant as part of a single study visit.

Once participants have provided consent, the following baseline data will be collected:

- Demographics (age, sex)
- Height, weight (to calculate body mass index)
- Skin assessment at RespiraSense device attachment site (color, texture, uniformity of appearance, integrity). It will also be confirmed that the skin has no discoloration or rash, epidermal loss, blistering, edema, tears, maceration or folliculitis
- Oxygen saturation and pulse oximetry

Participants will be asked to wear a hospital gown and be positioned on an examination couch in the simulation suite. The RespiraSense device, a nasal cannula, and an ECG will be attached and a video recorder positioned to ensure the viewers of the video experience the same image as the HCP performing manual RR monitoring.

A computer-based metronome will be used to produce a repetitive tone at a set rate, which is played to each participant via headphones. The metronome has been produced for the study by the study sponsor team using Microsoft Visual Basic. The metronome rates will be generated using a random number generator program, and the order of the different rates will vary with each participant to ensure the rates are random. The participants will be asked to begin to inspire when each tone is heard and will be given a 20-second warm-up period during which time they will regulate their breathing pattern to match the rate of the tone they hear. Each participant will be asked to breathe at one value from each of the following categories for 2 minutes:

- ≤8 breaths per minute
- 9 to 11 breaths per minute
- 12 to 20 breaths per minute
- 21 to 24 breaths per minute
- 25 to 29 breaths per minute
- 30 to 35 breaths per minute

One rate will be randomly generated from within each of the 6 categories. Examples of potential sets are shown in Table 2.

**Table 2:** Examples of possible sets of metronome rates to be used.

Participant	First rate	Second rate	Third rate	Fourth rate	Fifth rate	Sixth rate
Participant A	6	10	23	17	28	32
Participant B	5	14	9	22	31	26
Participant C	11	7	13	21	33	27

Each participant will be studied in the normal position (ie, sitting at 45 degrees on a couch) for 6 different metronome rates. They will then be studied in one of the three following additional positions for an additional 4 metronome rates: supine (flat on their back), left lateral decubitus (recovery) position, or sitting at 90 degrees (chair position).

**Figure 1:**
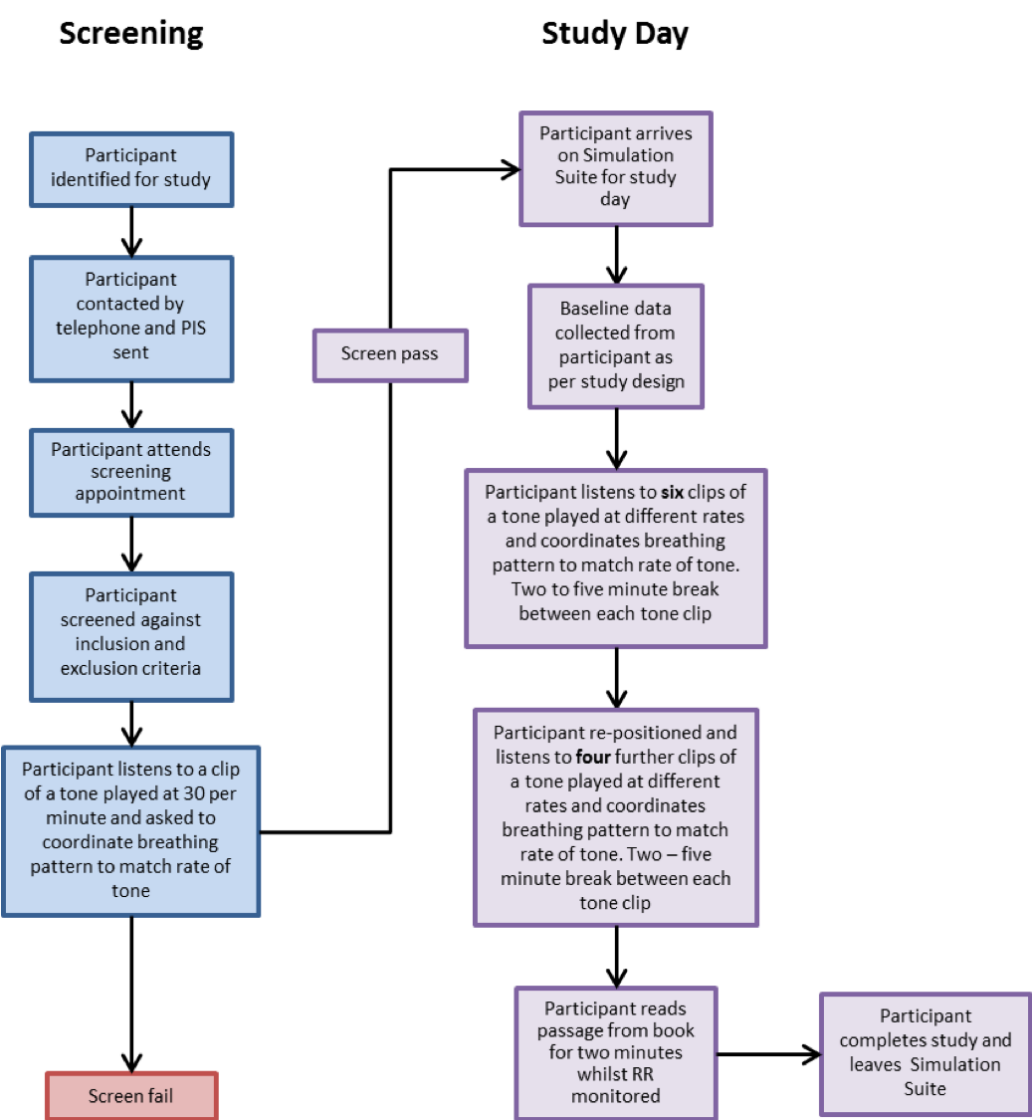
Participant process flow diagram.

Each metronome rate will be maintained for 2 minutes with 2 to 5 minutes in between for transition (or longer if required by the participant to return to normal breathing rates). A full process flow of the requirements of each participant is shown in Figure 1.

**Figure 2:**
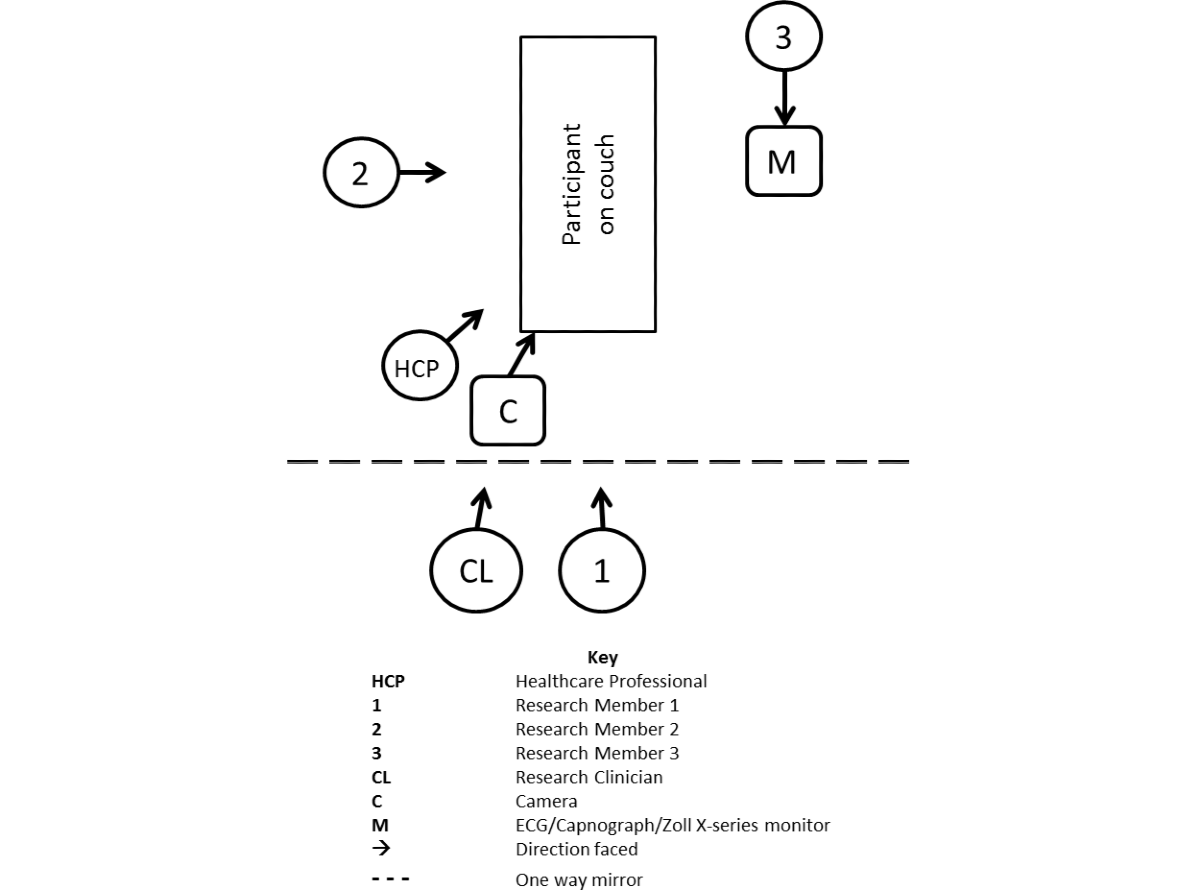
Schematic of room layout.

The research team will consist of 3 members; the layout of the study room is shown in Figure 2. Research member 1 will indicate to the participant when a new metronome rate starts. After the 20-second warm-up period, research member 1 will indicate the beginning of the 2-minute monitoring period by announcing the word Start. The HCP will start their manual counting of the participant’s RR 30 seconds after the beginning of each new metronome rate; they will be informed by research member 1 when to start counting. They will be given 60 seconds to calculate the manual RR and will be informed by research member 1 when to stop counting. They will then record the RR in the case report form. The participant will continue to breathe at the specified rate for a further 30 seconds (for a total of 2 minutes) and will be informed by research member 1 when the metronome rate has finished so that they return to normal breathing.

Research member 3, blinded to the rates being played in each metronome rate, will record RR values for the continuous ECG and capnography monitoring and the X Series monitor (Zoll Medical Corp) at 60 and 120 seconds. Research member 2 will press the Start and Stop button on the RespiraSense device at the beginning and end of each 2-minute metronome rate and will record the start and stop time on the case report form.

Following the 10 metronome rates, the participant will then be asked to read a verse from a book for 2 minutes. During this time, their RR will be monitored in the same way as for the 10 metronome rates. Once the participant has finished reading aloud, the study will finish.

Throughout the study procedure, a video recording will be made using the portable Scotia Medical Observation and Training System (Scotia UK Plc), the audiovisual system installed in the simulation suite, which includes an in situ camera system (hardware and software).

### Ethical Considerations

The study will not be initiated before the protocol and all study relevant material such as the informed consent forms and PIS have received approval/favorable opinion from the Health Research Authority and PHT Research and Development department. Any changes to protocol or relevant study documents will be approved by the sponsor. As RespiraSense is a CE-marked product being used within its intended purpose, regulatory approval is not necessary for this investigation.

### Study Device

The RespiraSense device is based on piezoelectric technology. This technology is in the form of films that are an ultraflat laminated layer of piezo material. This material, when bent or strained, produces a small varying voltage difference. This monitor measures the motion of the chest and abdomen during respiratory effort to measure the RR directly from this motion. The stages of development of these devices are described as fully compliant devices to European Medical Device Standards. RespiraSense is intended to act as a short-term monitoring device to assess RR over time by continuously recording, storing, and periodically transmitting RR data. It has no diagnostic function in this study.

Traceability of each device’s construction will be recorded in the company’s Design History File. Each lobe used on a patient will be recorded in an accountability log with the patient study number. Sensors will not be retained once used and will be disposed of by the patient or the HCP.

During this investigation, the sensor will be attached to the patient’s skin in the chest area using medical grade adhesive that has been tested to and complies with ISO10993 (Biological evaluation of medical devices). The RespiraSense device has the dimensions 100 mm × 55 mm × 15 mm, the sensor weighs 50 grams, and the following risks are listed:

- Class IIb medical device which conforms with IEC60601-1:A1:2012 and so no risk of electrical shock as no electrical current is passed through the body
- The device has low weight and as such is not considered to produce any skin ulcers
- The device will be adhered using medical grade tape which will minimize reaction to adhesive
- Wireless transmission of information conforms to standard Bluetooth communication protocols which are currently used in clinical settings
- Discomfort to the patient is considered minimum as the device in placed in a position where side-lying will not cause added pressure to the patient

**Figure 3:**
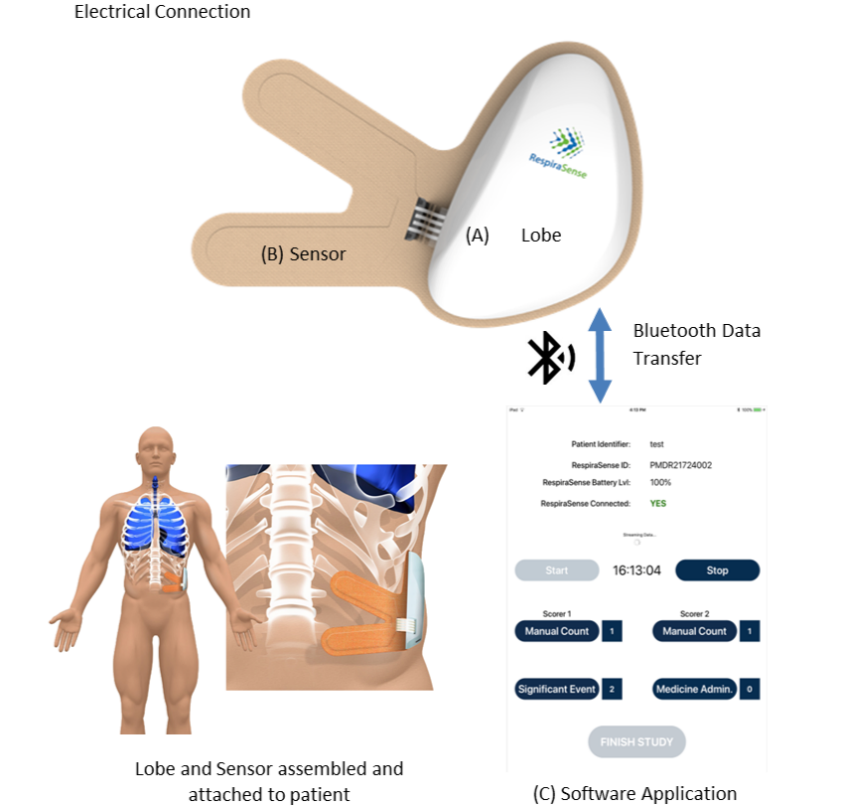
RespiraSense device and data flow.

The RespiraSense is a noninvasive, body-worn, cableless, battery-powered, respiration rate monitoring device. The system consists of 3 parts as shown in Figure 3: the lobe, sensor, and software application. This figure also shows the final assembled device attached to the patient.

The system uses a specifically designed adhesive attachment accessory (sensor), which fixes the device to the patient’s chest and contains piezo films that convert breathing motion into an electrical output. The lobe provides and communicates measurement values and technical information such as battery state wirelessly via Bluetooth to the software application hosted on a commercial off-the-shelf tablet computer. It can also provide basic information on activity status of the patient. The application and removal of the elements of the RespiraSense device, along with the maintenance of the device during operation, will be managed by a trained research nurse. Nasal end-tidal CO_2_ monitoring will be performed using Microstream Smart CapnoLine Plus EtCO_2_ sampling line (Zoll Medical Corp) with a disposable adult/intermediate nasal cannula. Data will be collected and automatically logged onto an SD card in the X Series monitor.

### Data Transfer and Processing

Once the participant has completed the study, all data collected by the RespiraSense device will be uploaded to a secure cloud-based storage system from which it will be extracted and analyzed by the team at PMD Solutions Ltd. This data will be anonymized and linked to a specific subject ID number allocated during the study. No participant identifiable data will be transmitted to the team at PMD Solutions Ltd. The RespiraSense-calculated RRs for each participant will then be matched to the data collected by the other methods for analysis.

The study database and video recording files will be stored on secure Trust electronic file servers. The associated file name storing this data will be labeled with the title of the clinical investigation and linked to the subject ID number. This file will be saved for a minimum duration of 5 years and will be made available to national authorities upon request in writing. All digital and paper archives will conform to the requirements of the Data Protection Act (United Kingdom, 2018) which incorporates the standards laid out by the General Data Protection Regulation (European Union, 2016).

### Statistical Analysis

The primary end point is the RR as measured by multiple methods. The primary objective of the analysis is to examine the agreement between the RespiraSense device and the other measurement methods for this end point. The mean difference between each pair of methods will be calculated with 95% confidence intervals. Variances will be tested for nonequality with an *F* test. The distributions of the differences will be examined with Bland-Altman plots. Limits of agreement (LoAs) and proportions of measurements outside the limits will be compared. If the differences between methods markedly increase with their mean, the analysis will be repeated with a log transformation of the measurements. A secondary analysis of agreement by participant position (45 degree sitting vs other positions) for each pair of methods will be performed using an analysis of variance.

### Patient Population Size

We expect to have statistically significant data following the collection of 300 data sets (10 distinct measurements taken from 30 unique participants). This is based on statistical analysis of previous studies.

The precision of the mean and precision of the LoAs depend on the standard deviation of the differences. We use the value of 1.79 breaths per minute as the standard deviation reported in the Lee study [12] for comparison of RespiraSense and ECG and the same value for the comparison of RespiraSense with end-tidal capnography, this being regarded as more accurate than ECG. For comparison with an HCP, we use the value of 2.5 breaths per minute, also from the Lee study.

Table 3 shows the number required for different precisions of the mean difference using 95% confidence intervals. Table 4 shows the number required for different precisions of the LoA.

**Table 3:** Precision of mean at 95% confidence interval.

Breaths per minute	Precision of the mean	Standard error of the mean	N
1.79	1	0.51	13
1.79	0.5	0.26	50
1.79	0.4	0.2	77
1.79	0.3	0.15	137
1.79	0.25	0.13	197^a^
1.79	0.2	0.1	308
1.79	0.1	0.05	1231
2.5	1	0.51	25
2.5	0.5	0.26	97
2.5	0.4	0.2	151
2.5	0.3	0.15	267^a^
2.5	0.25	0.13	385
2.5	0.2	0.1	601
2.5	0.1	0.05	2401

^a^Precisions to be expected if 30 participants provide 10 data points each, allowing for a 10% loss of data.

**Table 4:** Precision of limits of agreement at 95% confidence interval.

Breaths per minute	Precision of LoA^a^	Standard error of LoA	N
1.79	1	0.51	37
1.79	0.5	0.26	148
1.79	0.4	0.2	231^b^
1.79	0.3	0.15	411
1.79	0.25	0.13	591
1.79	0.2	0.1	924
1.79	0.1	0.05	3693
2.5	1	0.51	73
2.5	0.6	0.31	201^b^
2.5	0.5	0.26	289
2.5	0.4	0.2	451
2.5	0.3	0.15	801
2.5	0.25	0.13	1153
2.5	0.2	0.1	1801
2.5	0.1	0.05	7203

^a^LoA: limits of agreement.

^b^Precisions to be expected if 30 participants provide 10 data points each, allowing for a 10% loss of data.

### Feasibility Assessment

The feasibility of performing this study with data collected from participants in a single session is based on upon previous successful observational studies that have been performed by the research team at PHT. These include the Using the Inflammacheck Device to Measure the Level of Exhaled Breath Condensate Hydrogen Peroxide in Patients With Asthma and Chronic Obstructive Pulmonary Disease (EXHALE) pilot study [13] and a previous study validating the RespiraSense device in patients with a large body mass index [14].

## Results

Recruitment to this study has not yet started as funding decisions are still pending. Therefore, results are not available at this stage. It is anticipated that the data required could be collected within 2 months of first recruitment to the study and data analysis completed within 6 months of the study start date.

## Discussion

### Rationale for Study Design

The Evaluation of Agreement of Breathing Rates Measured by a Novel Device, Manual Counting, and Other Techniques Used in Clinical Practice (VENTILATE) study will test the accuracy of the RespiraSense device at the extremes of RR. The selection of randomized RRs from within defined categories allows these abnormal RRs to be compared with normal RRs without introducing variability in the participant or the HCP. Additionally, video recording of the study procedures allows for independent sampling and monitoring of the ability of the HCP to manually count the RR by another HCP.

We chose to use healthy volunteers without underlying cardiopulmonary disease. It was not deemed safe or appropriate to use patients exhibiting extremes of RR in an observational study of this type. It was decided that the safest way to observe the RRs of interest for the study would be for healthy volunteers to consciously breathe at a given rate.

There are multiple points of contact during the screening visit to minimize the chance that participants fail to complete the study procedures. In addition to the standard practice of providing a PIS and allowing the participant time to consider what will be required of them during the study, the participants will also experience one of the higher RRs that they will be asked to perform during the study day. We chose to do this with the aim of reducing the number of participants who are unable to complete all of the study procedures.

Although the risk of harm to study participants is low, further measures will be taken to mitigate these risks. The potential risk of feeling unwell after breathing at high or low RRs will be mitigated by allowing participants to rest for longer than scheduled or to stop the procedure entirely should they feel unable to continue. A clinician will be present throughout the study procedures and will be able to stop the study at their discretion if they feel it is not safe or would be inappropriate to continue.

Additionally, the randomization of the RRs selected will be prescreened to prevent high variability between tachypnoea and bradypnea in a short space of time. A set of rates will be randomly generated by a custom random number generator which is programmed to select a rate from within each of the categories listed in the Study Design section. Each set generated will be reviewed by a clinician involved in the study and assessed for extreme variability and overall safety. Sets that pass this screening will be saved and assigned randomly to participants at the time of the study data collection. Examples of an acceptable and unacceptable set are shown in Table 5.

**Table 5:** Acceptable and unacceptable respiratory rate sets.

	First rate	Second rate	Third rate	Fourth rate	Fifth rate	Sixth rate
Acceptable	6	10	23	17	28	32
Unacceptable	5	31^a^	22	14	26	9^a^

^a^Rates with extreme variability.

Another potential risk for participants using the RespiraSense device is the risk of reaction to the adhesive. This risk has two parts. The first is allergic reaction to the adhesive. This risk has been given a risk level of 2 by the PMD risk management team. A risk level of 2 indicates the risk has been reduced as low as possible. Although the severity of this potential risk is high, this occurrence has not been observed by PMD in any use of the device and has not been found in literature searches. This risk is mitigated by excluding volunteers who are allergic to medical-grade adhesive.

The second component is a mild reaction to the adhesive. This has been given a risk level of 2 by the PMD risk management team. A risk level of 2 indicates the risk has been reduced as low as possible. This risk is of low severity (mild reaction/discomfort) and moderate occurrence level. This risk is mitigated by excluding volunteers based on a skin fragility assessment. It is also possible that mild irritation that will not require medical intervention may be experienced for patients on oral steroids.

The procedures will be carried out in the simulation suite in the Learning and Development department which is located within the Resuscitation Department of PHT, and an experienced member of the resuscitation team will always be present. If a participant should become unwell as a result of the procedures, they will be withdrawn from the study and assessed by clinical team, and subsequently care will be escalated as appropriate.

The outputs of the RespiraSense sensor will not be used to assist any clinical decision, so there are no risks associated with use of the data collected with the device in this study.

### Limitations

The primary limitation to this study is the awareness from all participants that they are being monitored. This may alter the behavior of the participant and subsequently influence the data collected from them, known as the Hawthorne effect. The most likely influence that is expected to be seen is a change in the method of manual RR counting performed by the HCP. There is a large degree of variability in clinical practice when counting the RR. The awareness of being observed has the potential to encourage those who would use a method involving a shorter count and multiplication to employ a full count for 60 seconds. This will reduce the accuracy of the comparison between the RespiraSense device and current clinical practice.

### Conclusions

The VENTILATE study will provide further validation of the use of the RespiraSense device in subjects with abnormal respiratory rates. It will assess the device’s ability to measure abnormal RRs and compare with multiple other techniques used in various aspects of clinical practice. The results provided will help to guide the usefulness of the device in clinical practice in the future.

## Abbreviations

ECG: electrocardiogram
EXHALE: Using the Inflammacheck Device to Measure the Level of Exhaled Breath Condensate Hydrogen Peroxide in Patients With Asthma and Chronic Obstructive Pulmonary Disease pilot study
HCP: health care provider
LoA: limit of agreement
NEWS: National Early Warning Score
NICE: National Institute for Clinical Excellence
PHT: Portsmouth Hospitals NHS Trust
PIS: participant information sheets
RR: respiratory rate
VENTILATE: Evaluation of Agreement of Breathing Rates Measured by a Novel Device, Manual Counting, and Other Techniques Used in Clinical Practice
